# The Best Education

**Published:** 1872-06

**Authors:** 


### THE BEST EDUCATION

Is not that which imparts to our children at school the most of grammar and geography and mathematics and general literature; these are mere acquisitions. He is the best educator who most successfully teaches his pupils how to think for themselves, and who most deeply impresses on their minds the importance of generous purposes, of high aspirations and exalted ambitions, with an unalterable determination to live for humanity, for God, for immortality. But to do these things most effectually, the young should be early taught the duty of caring for the body as conscientiously as they care for the soul; for the mind works best through a healthy brain.

